# Impact of Screening on Breast Cancer Incidence in Kazakhstan: Results of Component Analysis 

**DOI:** 10.31557/APJCP.2021.22.9.2807

**Published:** 2021-09

**Authors:** Asem Toguzbayeva, Zhansaya Telmanova, Arman Khozhayev, Aizhan Jakipbayeva, Gulshara Aimbetova, Akmaral Zhantureyeva, Zarina Bilyalova, Saltanat Urazova, Gulnur Igissinova, Botagoz Turdaliyeva, Malcolm Anthony Moore, Nurbek Saginbekovich Igissinov

**Affiliations:** 1 *Asfendiyarov Kazakh National Medical University, Almaty, Kazakhstan. *; 2 *Central Asian Cancer Institute, Nur-Sultan, Kazakhstan. *; 3 *Astana Medical University, Nur-Sultan, Kazakhstan. *; 4 *Kazakh Medical University of Continuing Education, Almaty, Kazakhstan. *; 5 *Eurasian Institute for Cancer Research, Bishkek, Kyrgyzstan. *

**Keywords:** Breast cancer, incidence, screening, component analysis, Kazakhstan

## Abstract

**Objective:**

The study is to conduct a component analysis of the dynamics of the incidence of BC (BC) in Kazakhstan, taking into account regions.

**Methods:**

Primary data were for registered patients with BC (ICD 10 – C50) in the whole country during the period of 2009-2018. Evaluation of changes in BC incidence in the population of Kazakhstan was performed using component analysis according to the methodological recommendations.

**Results:**

The study period, 40,199 new cases of BC were recorded. The incidence rate increased from 39.5 (2009) to 49.6 in 2018 and the overall growth was 2.8 per 100,000 population of female, including due to the age structure – ∑Δ_A_=+2.99, due to the risk of acquiring illness – ∑Δ_R_=+6.82 and their combined effect – ∑Δ_RA_=+0.31. The component analysis revealed that the increase in the number of patients with BC was mainly due to the growth of the population (Δ_P_=+31.1%), changes in its age structure (Δ_A_=+18.0%) and changes associated with the risk of acquiring illness (Δ_R_=+41.0%). The increase in the number of patients in the regions of the republic is associated with the influence of demographic factors and with risk factors for getting sick, including mammographic screening.

**Conclusion:**

Thus, as a result of the component analysis, the role of the influence of demographic factors and the risk of acquiring illness on the formation of the number of patients and the incidence of BC was evaluated, while geographical variability was established. This research was the first epidemiological study of the dynamics of BC in the regional context by the method of component analysis in the population of Kazakhstan. The implementation of the results of this study is recommended in management of anticancer activities for BC.

## Introduction

Despite significant advances in research, BC remains a major public health issue and is a priority for biomedical research. The incidence of BC, according to the International Agency for Research on Cancer, is about 2.3 million new cases annually, and the age-standardized incidence rate (ASR) is 47.8 per 100,000 population of female (Ferlay et al., 2020A) and remains alarmingly high; these figures indicate slow progress in prevention (DeSantis et al., 2011; DeSantis et al., 2014; Kolak et al., 2017; Winters et al., 2017).

Worldwide, BC is the most common type of cancer affecting women, and its incidence and mortality are expected to increase significantly in the next 5-10 years (Greaney et al., 2015). According to IARC, more than 3 million cases of BC are expected by 2040, about 1.4 million cases of which are among the Asian population, with an increase of 38% in this region (Ferlay et al., 2019; Ferlay et al., 2020B). Therefore, the most important element of modern medicine is conducting interdisciplinary research aimed at improving the effectiveness of disease prevention by focusing on primary prevention, modifying risk factors for early detection of the disease, rapid initiation of treatment (secondary prevention), as well as medical supervision. The main goal is to reduce the ever-increasing morbidity, mortality, and economic costs of BC (Howell et al., 2014; Coughlin et al., 2015; Sun et al., 2017; Thorat et al., 2020; Britt et al., 2020).

More developed countries account for half of all cases and 38% of deaths from BC (Curado et al., 2007; Torre et al., 2012). Early detection and different risk factors may explain the differences in the variability of BC in the world. At the same time, various genetic factors (Calderon-Margalit and Paltiel, 2004; Shah et al., 2009) and environmental factors (Wolff et al., 2003; Song et al., 2011), especially co-existing ones, increase the risk of BC. Environmental and lifestyle factors include the followings: hormone therapy (Russo and Russo, 2007), reproductive behavior (Russo and Russo, 2000; Jacobson et al., 2008), alcohol intake (Nagykálnai and Landherr, 2018; Freudenheim, 2020), and other dietary factors (Harvie et al., 2015; Sellami and Bragazzi, 2020), obesity (Pettapiece-Phillips et al., 2015), and physical inactivity (Podkowa et al., 2014; Howell et al., 2014; Coughlin et al., 2015; de Boer et al., 2017). Other commonly recognized health-socio-demographic risk factors include age and the burden of cancer in family, especially the burden of BC (Kotsopoulos et al., 2005; Kruk, 2012).

In Kazakhstan, mammological screening was conducted from 2008-2016, when the contingent of persons for the study were women 50 years and older. BC screening decisions should always be individual, taking into account the patient’s risk of developing the disease, their values and preferences, and a full discussion of the potential benefits and harms of testing. Studies indicate screening in women starting at the age of 40, but not all support this recommendation (Tonelli et al., 2011; Wilt et al., 2015; Siu, 2016). A person’s overall health and life expectancy should be taken into consideration when deciding on the age of discontinuation of BC screening. For women over the age of 75, cancer may be diagnosed at an earlier stage, but may not be the cause of reduced mortality in part due to shorter life expectancy (Smith-Bindman et al., 2000; Braithwaite et al., 2016). In addition, epidemiological studies on BC have shown that there is an ethnic difference in the average age of patients (Bilyalova, 2012), so the Kazakh women – 50 years, and Russian women – 60 years. After the revision of the age of the contingent subject to screening since 2017, it is carried out in the republic from the age of 40.

Anti-cancer measures have a significant impact on the formation of oncological indicators, including morbidity and mortality. The study of the influence of demographic factors, risk factors, considering the exogenous and endogenous ones, is a priority task of oncoepidemiology. The aim of presenting study is to assess the impact of various factors by using a component analysis of the dynamics of incidence.

## Materials and Methods

*Cancer registration and patient recruitment*

The population of republic of Kazakhstan as the 2018 census was 18.2 million, of which 9.36 million were females (Bureau of National Statistics, 2018). The cancer registry of the population of Kazakhstan covers 14 regions and cities of national significance-Almaty and Astana (now the city of Nur-Sultan). New cases of BC were extracted from the accounting and reporting forms of the Ministry of Health of the Republic of Kazakhstan – form 7 and form 35, which were formed from the register of oncological diseases based on the administrative-territorial division of the republic from 2009 to 2018 using the International Disease Code 10, code C50.

*Population denominators*

Population denominators for calculation of incidence rates were provided by the Bureau of National Statistics for 2009-2018. At the same time, data on the number of female population of the republic, taking into account the studied regions, are used, all data are presented on the official website (www.stat.gov.kz).

*Statistical analysis*

The main method used in the study of incidence was a retrospective study using descriptive and analytical methods of modern oncoepidemiology. Age-standardized incidence rates (ASRs) were calculated for eighteen different age groups (0-4, 5-9, …, 80-84, and 85+) and ten calendar periods from 2009 to 2018 (1-year intervals). ASRs standardized to the world population proposed by World Health Organization (Ahmad et al., 2001) with recommendations from the National Cancer Institute (http://seer.cancer.gov/stdpopulations/world.who.html) were estimated for each studied year.

The extensive, crude and age-specific incidence rates (ASIR) are determined according to the generally accepted methodology used in modern sanitary statistics. The annual averages (M, P), mean error (m), Student criterion, 95% confidence interval (95% CI), and average annual upward/downward rates (T%) were calculated. We did not justify the main calculation formulas in this paper, since they are detailed in the methodological recommendations and textbooks on medical and biological statistics (Merkov and Polyakov, 1974; Glanc, 1999; dos Santos Silva, 1999). 

The dynamics of incidence rates was studied for 10 years, while the trends of incidence were determined by the least squares method. To calculate the average annual growth rate and/or growth rate of the dynamic series, the geometric mean equal to the root of the power of n from the product of the annual growth rate indicators was used.

The dynamics of the incidence of BC was studied using a component analysis according to the methodological recommendations (Dvoyrin and Aksel, 1987). The method of component analysis was used in this study to break down the growth of number of cases belonging to the same population, but in different time periods.

There are 7 components of the increase in the number of cases. The first 3 components are related to changes in the population number, its age structure, and the combined influence of these factors. The true increase in the number of patients with oncological pathology is due only to a change in the risk indicator of morbidity and is represented by the 4th component. The following 3 components are associated with the risk of developing a malignant neoplasm, with the growth of the population, changes in its age structure, and the influence of all three factors. Thus, the last 4 components are associated with an increase in the risk of developing the disease. The “risk of acquiring illness” refers to the whole range of reasons that can lead to an increase, decrease or stabilization of morbidity rates.

The method of components was applied to study the dynamics of the number of BC patients and has been performed on cases that occurred from 2009 to 2018 among the entire population of the country. Assessments of the component analysis of the dynamics of morbidity of BC in the population of Kazakhstan are presented in the relevant tables.

Viewing and processing of the received materials was carried out using the Microsoft 365 software package (Excel, Word, PowerPoint), in addition, online statistical calculators were used (https://medstatistic.ru/calculators/averagestudent.html), where Student criterion was calculated when comparing the average values.

*The following symbols and abbreviations were used in this article*

AN, absolute number; ASIR, age specific incidence rate; ASP (Δ_A_), the age structure of the population; ASR, age-standardized rate; END, the expected number of diseases; NCRC, the number of BC cases; PN (Δ_P_), population number; RAI (Δ_R_), risk of acquiring illness; R^2^, the value of the approximation confidence; SI, structural indexes; Р, the incidence of BC; ^0^/_0000_ – prosantimille, designation per 100,000.

*Ethics approval*

Because this study involved the analysis of publicly available administrative data and did not involve contacting individuals, consideration and approval by an ethics review board was not required. At the same time, the submitted data is in accordance with the Law of the Republic of Kazakhstan No. 257-IV of March 19, 2010 “About State statistics” (http://adilet.zan.kz/rus/docs/Z100000257), the information in the summary report is confidential and can only be used for statistical purposes in accordance with the Principles of the World Medical Association (WMA, 2013).

## Results

During the study period, 40,199 new cases of BC were registered in the republic. The greatest proportion of patients falls on the age of 50-59 years (50-54 years – 15.6% and 55-59 years – 16.1%) (Table 1).

Age-related indicators of BC incidence had a peak in 65-69 years (167.2±12.1^0^/_0000_). Trends in age-related indicators of BC incidence tended to increase in almost all age groups, except for group of 20-24 years (T=−1.0%), 25-29 years (T=−1.6%) and 85 and older (T=−1.8%), where there was a decrease. It should be noted that the value of the approximation confidence of the listed decreases is not significant (Table 1).

Trends in age-related indicators affected the overall incidence rates, so the crude incidence rates of BC increased from 39.5 (2009) to 49.6 per 100,000 female population in 2018 (p=0.000), the total increase was 10.13^0^/_0000_ (Table 2) and depended on changes in the age structure of the population (∑Δ_A_=+2.99^0^/_0000_), the risk of acquiring illness (∑Δ_R_=+6.82^0^/_0000_) and the combined influence of the age structure and the risk of acquiring illness (∑Δ_AR_=+0.31^0^/_0000_). At the same time, the average annual growth rate of the aligned indicator was T=+2.8%, and the value of the approximation confidence was close to 1 (R^2^=0.843; Table 2).

Then, we will consider the component analysis results of the dynamics of the number of patients with BC in the republic as a whole (Table 2). The results of the study show that the growth in the number of patients with BC in the republic was associated with the influence of the following factors:

1. Growth of population number Δ_P_=+31.1%.

2. Changes in the age structure of the population Δ_A_=+18.0%.

3. Combined effect of changes in population number and its age structure Δ_PA_=+2.4%.

4. Risk of acquiring illness Δ_R_=+41.0%.

5. Combined effect of changes in the risk of acquiring illness and population number Δ_PR_=+5.4%.

6. Combined effect of changes in the risk of acquiring illness and age structure of the population Δ_RA_=+1.9%.

7. Combined effect of the changes in the risk of acquiring illness of the population and its age structure Δ_RAP_=+0.2%.

The total increase in the absolute number of patients overall equals the sum of components:

*n*_2_ *−n*_1_=428+248+32+565+74+28+3=1376 or +42.1% in comparison with the primary number of patients (1376÷3272×100=42.1%).

At the same time, the components of the increasing in the percentage at the primary level are equal for the women population:



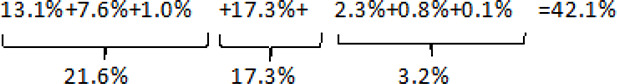



Thus, BC is characterized by an increase in the number of cases as a result of changes in the total number and structure of the female population (21.6% of the total increase of 42.1%). The real increase in the number of cases (risk of acquiring illness) was Δ_R_ =+17.3%.

In the dynamics of the incidence of BC had regional characteristics. Thus, in the Karaganda region, the overall increase in the incidence of BC was the highest and amounted to +28.37 per 100,000 female population, increased from 44.36^0^/_0000_ (2009) to 72.73^0^/_0000_ in 2018 (p=0.000) (Table 2) and depended primarily on the risk of acquiring illness (∑Δ_R_=+22.69^0^/_0000_), and secondly, on changes in the age structure of the population (∑Δ_A_=+3.13^0^/_0000_), and thirdly on the combined influence of age structure and risk of acquiring illness (∑Δ_RA_=+2.56^0^/_0000_). At the same time, the average annual growth rate of the aligned indicator was T=+4.1%, and the value of the approximation confidence was R^2^=0.778. Analyzing the role of various components, it was found (Table 2) that the increase of patients in this area is associated with demographic factors (Δ_P+A+PA_=+14.3%) and the complex influence of the risk of acquiring illness (Δ_R_=+75.0%) with the components of the population number, its age structure, and the influence of all three of the above factors (Δ_R+PR+RA+RAP_=+85.6%).

While analyzing the average annual growth rate of the aligned indicators of the incidence of BC, it was found that the largest increase was in the Mangystau region (T=+7.1%; R^2^=0.508), while the growth in 2018 was statistically significant compared with 2009, and the values of the approximation confidence were pronounced.

Analyzing the results of the influence of various components by region (Table 2), it was found that due to changes in the population, there is a pronounced decrease in the North Kazakhstan region (Δ_P_=−23.9%) and the largest increase in Almaty (Δ_P_=+127.7%) and in Astana (Δ_P_=+97.0%). The role of the influence of age structure in the rise in the number of patients was positive in all regions, but most pronounced was in Pavlodar (Δ_A_=+126.0%) and East Kazakhstan (Δ_A_=+85.2%) regions. The combined effect of changes in the population number and its age structure showed a decline only in North Kazakhstan (Δ_PA_=−2.7%), East Kazakhstan (Δ_PA_=−1.2%), Kostanay (Δ_PA_=−0.4%) and Akmola (Δ_PA_=−0.2%) regions, while in the other regions there was an increase – especially in South Kazakhstan (Δ_PA_=+3.6%) and Kyzylorda (Δ_PA_=+3.3%) regions, as well as in Astana city (Δ_PA_ =+8.4%). The reduction in the absolute number of patients with BC due to the risk of acquiring illness was most pronounced in Almaty (Δ_R_=−25.7%), and the maximum increase was established in North Kazakhstan (Δ_R_=+97.5%), Karaganda (Δ_R_=+75.0%), West Kazakhstan (Δ_R_=+69.6%), Akmola (Δ_R_=+67.9%) and Kostanay (Δ_R_=+61.6%) regions. A pronounced growth in the combined impact of the risk of acquiring illness and the population number was found in the Mangystau (Δ_PR_=+15.4%) and Atyrau (Δ_PR_=+10.9%) regions. And a pronounced decrease was found in Almaty (Δ_PR_=−8.5%). Changes in the risk of acquiring illness and the age structure led to a sharp decrease in the number of patients in the Pavlodar region (Δ_RA_=−25.4%), and the maximum increase was noted in the Kostanay region (Δ_RA_=+20.2%). The increase in patients due to the combined influence of the risk of acquiring illness, population size and age structure was the highest in South Kazakhstan and Mangystau regions (Δ_RAP_=+1.7%) compared to other regions (Table 2).

Next, we will review the regional features of the incidence of BC, taking into account the age of women to be screened. Thus, the incidence in the age group 40-69 years (Table 3) increased from 98.3±2.0 (2009) to 119.3±2.0 (p=0.000) per 100,000 female population, and the overall increase (+20.94^0^/_0000_) was mainly due to the risk of acquiring illness (∑Δ_R_=+14.34^0^/_0000_), and the average annual growth rate of the aligned indicator was Тg=+2.6% and the value of the approximation confidence is R2=0.7486.

Analyzing Table 3, we would like to focus on changes in the incidence of BC among women 40-69 years old at the regional level. Hence, the highest overall increase in morbidity was found in the West Kazakhstan region (+61.29^0^/_0000_) and was also associated with the risk of acquiring illness (∑Δ_R_=+53.42^0^/_0000_), while the growth in the number of patients 40-69 years old was due to the influence of this factor (Δ_R_=+63.1%). A high increase in the number of patients due to this factor was found in the Karaganda (Δ_R_=+71.0%) and North Kazakhstan (Δ_R_=+73.3%) regions (Table 3).

A decrease was found in the cities of Astana (−9.39^0^/_0000_) and Almaty (−13.72^0^/_0000_) (Table 3), which was primarily associated with a reduction of the risk of acquiring illness (∑Δ_R_=−20.75^0^/_0000_ и ∑Δ_R_=−19.30^0^/_0000_, respectively), but the trends in incidence tended to rise (Т=+1.6% и Т=+1.4%, respectively), but the approximation value was not significant in both cases. It should be noted that during the study period in these cities, the number of patients with BC in the target group due to the influence of the risk of acquiring illness would have decreased by Δ_R_=−18.2% and Δ_R_=−56.6%, respectively, and the increase in the number of patients in the cities of Astana (Δ_P_=+114.9%) and Almaty (Δ_P_=+154.5%) was associated with an growth of the female population (Table 3). Also, the number of patients in the East Kazakhstan (Δ_R_=−19.7%) and Pavlodar (Δ_R_=−26.3%) regions would decrease due to this factor (changes in the risk of acquiring illness).

In order to exclude the influence of the age structure of the female population of the regions, standardized indicators of incidence and mortality were calculated. Thus, the ASR in the republic as a whole was 42.6±1.0 (95% CI=40.7-44.5) per 100,000 female population, the dynamics of the indicators tended to grow (p=0.000) and the average annual growth rate of the aligned indicator was Т_g_=+2.0, and the value of the approximation confidence R^2^=0.7277.

High ASR were found in Karaganda (47.9) and Pavlodar (51.5) regions, as well as in Astana (61.2) and Almaty (61.9) cities. Low ASR were detected in South Kazakhstan (30.1), Kyzylorda (30.4) and Zhambyl (31.4) regions (Table 4).

The analysis of the trends of the aligned standardized indicators of the incidence of BC showed that the highest average annual growth rates were established in the Atyrau (Т_g_=+4.0) and Mangystau (Т_g_=+6.1) regions.

In dynamics, the standardized incidence rates from BC in Kazakhstan tended to decrease from 16.0±0.4 (2009) to 10.9±0.3 per 100,000 female population in 2018 (p=0.000), and the average annual incidence rate for the studied years was 13.9±0.6 (95% CI=12.7-15.0). The average annual rate of decline of the aligned indicator was Т_d_=−4.0% (R^2^=9218). High standardized mortality rates from BC were established in Zhambyl (15.0) and Pavlodar (16.9) regions, as well as in the cities of Almaty (19.2) and Astana (19.3) (Table 4). Trends in the aligned standardized mortality rates from BC lowered in almost all regions of the country, apart from the Atyrau region, where there was an increase (T_g_=+1.7%). The highest rates of decline were found in Karaganda (Т_d_=−7.4%, R^2^=0.7792) and East Kazakhstan (Т_d_=−7.5%, R^2^=0.7707), while the values of the approximation confidence were pronounced (Table 4).

Hereby, the component analysis revealed geographical variability in the dynamics of the number of patients and of the incidence of BC in Kazakhstan, which were associated with a difference in the influence of demographic factors (changes in population number, age structure) and the risk of acquiring illness, i.e. a set of reasons that led to an increase, decrease or stabilization of the incidence rates. Upon the whole, there is an increase in incidence and a decrease in mortality from BC in the republic.

**Table 1:** Breast Cancer in Kazakhstan, 2009-2018

Age	Number %	Incidence			
		per 100,000		Т, %	R^2^
		P±m, ^0^/_0000_	95% CI		
15-19	8 (0.02)	0.13±0.05	0.04-0.22	+6.9	0.0332
20-24	67 (0.17)	0.88±0.19	0.51-1.24	−1.0	0.0024
25-29	308 (0.77)	4.0±0.3	3.4-4.6	−1.6	0.0408
30-34	950 (2.4)	14.0±0.6	12.7-15.3	+3.1	0.4452
35-39	1803 (4.5)	29.5±1.0	27.6-31.4	+2.2	0.4268
40-44	3141 (7.8)	54.8±2.1	50.7-58.9	+2.8	0.5179
45-49	4732 (11.8)	84.8±1.8	81.3-88.2	+1.4	0.4414
50-54	6265 (15.6)	116.4±4.7	107.2-125.5	+1.4	0.1216
55-59	6470 (16.1)	142.6±3.7	135.3-150.0	+0.2	0.0085
60-64	5503 (13.7)	158.8±2.7	153.5-164.2	+1.0	0.3127
65-69	4056 (10.1)	167.2±12.1	143.5-190.8	+6.3	0.7011
70-74	3076 (7.7)	149.5±5.3	139.1-159.9	+2.9	0.6467
75-79	2281 (5.7)	138.2±4.7	129.0-147.5	+1.3	0.1458
80-84	1031 (2.6)	114.0±5.4	103.4-124.5	+2.7	0.3123
85+	508 (1.3)	92.0±4.6	83.0-101.1	−1.8	0.1291
Total	40199 (100.0)	45.4±1.4	42.8-48.1	+2.8	0.8427

**Table 2:** Component Analysis of Breast Cancer Incidence by Regions of Kazakhstan, 2009-2018

Regions	Incidence, ^0^/_0000_		Incidence growth, ^0^/_0000_				T, %	p	R^2^	AN	Change/ Combined, %							Total
			general*	Including							Δ_P_	Δ_A_	Δ_PA_	Δ_R_	Δ_RP_	Δ_RA_	Δ_RAP_	
	2009	2018		Δ_A_	Δ_R_	Δ_RA_												
Almaty city	62.48	59.12	−3.36	+1.32	−4.16	−0.52	+1.0	0.375	0.043	119	+127.7	+8.1	+2.7	−25.7	−8.5	−3.2	−1.1	100.0
Astana city	55.39	56.11	+0.72	+4.81	−3.59	−0.50	+2.0	0.895	0.246	129	+97.0	+11.5	+8.4	−8.6	−6.3	−1.2	−0.9	100.0
Kyzylorda	24.37	29.42	+5.05	+2.13	+2.26	+0.66	+2.7	0.192	0.583	32	+38.3	+22.7	+3.3	+24.1	+3.6	+7.0	+1.0	100.0
South Kazakhstan	20.67	25.90	+5.22	+1.99	+2.32	+0.92	+4.1	0.005	0.674	123	+37.6	+20.2	+3.6	+23.5	+4.2	+9.3	+1.7	100.0
Pavlodar	63.07	69.90	+6.83	+9.61	−0.85	−1.94	+1.5	0.242	0.189	30	+9.5	+126.0	+1.4	−11.1	−0.1	−25.4	−0.3	100.0
Zhambyl	26.13	33.83	+7.69	+2.58	+4.27	+0.85	+2.5	0.019	0.495	55	+20.6	+24.6	+2.0	+40.7	+3.4	+8.1	+0.7	100.0
East Kazakhstan	54.63	62.71	+8.08	+6.15	+2.48	−0.56	+1.8	0.041	0.479	53	−10.3	+85.2	−1.2	+34.3	−0.5	−7.7	+0.1	100.0
Kazakhstan	39.50	49.63	+10.13	+2.99	+6.82	+0.31	+2.8	0.000	0.843	1376	+31.1	+18.0	+2.4	+41.0	+5.4	+1.9	+0.2	100.0
Almaty	25.88	36.99	+11.11	+2.29	+7.81	+1.01	+4.5	0.000	0.394	139	+18.6	+15.2	+1.6	+51.7	+5.6	+6.7	+0.7	100.0
Aktobe	34.12	47.00	+12.88	+4.10	+9.97	−1.19	+1.4	0.004	0.198	74	+22.9	+21.8	+2.8	+52.9	+6.7	−6.3	−0.8	100.0
Atyrau	24.22	38.10	+13.87	+2.67	+11.33	−0.13	+4.9	0.003	0.573	57	+23.3	+12.2	+2.6	+51.7	+10.9	−0.6	−0.1	100.0
Kostanay	48.68	64.08	+15.40	+3.70	+8.81	+2.89	+2.7	0.002	0.300	67	−5.8	+25.9	−0.4	+61.6	−1.1	+20.2	−0.3	100.0
Akmola	44.36	60.15	+15.79	+4.23	+10.46	+1.10	+4.0	0.003	0.598	59	−1.9	+27.5	−0.2	+67.9	−0.4	+7.1	0.0	100.0
Mangistau	23.38	39.83	+16.45	+1.79	+13.20	+1.46	+7.1	0.001	0.508	75	+27.3	+5.8	+2.1	+42.9	+15.4	+4.8	+1.7	100.0
North Kazakhstan	60.04	82.97	+22.93	+6.71	+16.82	−0.59	+2.9	0.001	0.653	54	−23.9	+38.9	−2.7	+97.5	−6.7	−3.5	+0.2	100.0
West Kazakhstan	39.37	65.34	+25.97	+4.40	+21.57	−0.01	+3.9	0.000	0.534	96	+9.7	+14.2	+1.1	+69.6	+5.3	0.0	0.0	100.0
Karaganda	44.36	72.73	+28.37	+3.13	+22.69	+2.56	+4.1	0.000	0.778	214	+3.7	+10.3	+0.3	+75.0	+1.9	+8.5	+0.2	100.0

*The table is built taking into account the sorting from A to Z of the general growth.

**Table 3:** Component Analysis of Breast Cancer Incidence in Women Aged 40-69 Years by Regions of Kazakhstan, 2009-2018

Regions	Incidence, ^0^/_0000_		Incidence growth, ^0^/_0000_				T, %	p	R^2^	AN	Change/ Combined, %							Total
			general*	Including							Δ_P_	Δ_A_	Δ_PA_	Δ_R_	Δ_RP_	Δ_RA_	Δ_RAP_	
	2009	2018		ΔA	Δ_R_	Δ_RA_												
Almaty city	148.83	135.11	−13.72	+4.16	−19.30	+1.41	+1.4	0.190	0.0450	77	+154.5	+12.2	+4.3	−56.6	−20.0	+4.1	+1.5	100.0
Astana city	161.47	152.08	−9.39	+11.34	−20.75	+0.02	+1.6	0.591	0.1380	92	+114.9	+9.9	+8.1	−18.2	−14.8	±0.0	±0.0	100.0
Pavlodar	130.62	136.42	+5.80	+10.35	−4.96	+0.42	+1.3	0.669	0.1227	26	+66.3	+54.8	+5.3	−26.3	−2.5	+2.2	+0.2	100.0
East Kazakhstan	116.55	123.70	+7.14	+9.65	−3.08	+0.57	+1.1	0.454	0.2624	40	+51.2	+61.7	+4.2	−19.7	−1.4	+3.6	+0.2	100.0
Zhambyl	73.73	87.66	+13.93	+3.00	+9.44	+1.49	+2.2	0.170	0.3944	43	+45.3	+9.9	+1.8	+31.3	+5.8	+4.9	+0.9	100.0
Kyzylorda	72.94	88.05	+15.11	+2.03	+13.24	−0.17	+2.6	0.234	0.5733	34	+50.5	+5.2	+1.4	+34.2	+9.2	−0.4	−0.1	100.0
South Kazakhstan	65.15	83.77	+18.62	+2.22	+13.83	+2.57	+4.0	0.005	0.6752	125	+44.3	+5.1	+1.5	31.9	+9.4	+5.9	+1.7	100.0
Kostanay	104.30	123.53	+19.23	+5.06	+8.26	+5.92	+2.3	0.099	0.2114	44	+24.3	+18.8	+1.2	+30.6	+1.9	+21.9	+1.4	100.0
Almaty	69.37	89.21	+19.84	+3.60	+15.85	+0.39	+3.8	0.007	0.3145	99	+36.9	+9.5	+1.9	+42.0	+8.4	+1.0	+0.2	100.0
Kazakhstan	98.33	119.28	+20.94	+5.53	+14.34	+1.08	+2.6	0.000	0.7486	1108	+43.9	+12.3	+2.5	+32.0	+6.4	+2.4	+0.5	100.0
Akmola	101.22	127.54	+26.32	+4.44	+16.45	+5.43	+3.4	0.043	0.5355	47	+21.0	+12.4	+0.9	+46.0	+3.4	+15.2	+1.1	100.0
Aktobe	87.05	118.32	+31.27	+7.40	+25.62	−1.74	+0.9	0.014	0.0858	66	+34.3	+12.6	+2.9	+43.7	+10.1	−3.0	−0.7	100.0
North Kazakhstan	125.49	158.77	+33.28	+5.25	+26.02	+2.00	+1.1	0.033	0.1075	41	+4.9	+14.8	+0.2	+73.3	+1.0	+5.6	+0.1	100.0
Atyrau	63.62	104.27	+40.65	+8.63	+32.07	−0.06	+4.9	0.006	0.6082	49	+26.0	+12.2	+3.5	+45.3	+13.1	−0.1	±0.0	100.0
Mangistau	78.07	127.32	+49.25	+2.50	+46.29	+0.47	+6.6	0.004	0.4624	64	+32.9	+2.3	+1.1	+43.5	+19.5	+0.4	+0.2	100.0
Karaganda	91.63	151.78	+60.14	+4.37	+52.08	+3.69	+4.1	0.000	0.7875	177	+10.9	+6.0	+0.5	+71.0	+6.2	+5.0	+0.4	100.0
West Kazakhstan	91.66	152.95	+61.29	+8.19	+53.42	−0.32	+3.9	0.000	0.4593	84	+16.5	+9.7	+1.5	+63.1	+9.6	−0.4	−0.1	100.0

*The table is built taking into account the sorting from A to Z of the general growth.

**Table 4:** Evaluation of Standardized Indicators of Incidence and Mortality from BC by Regions of Kazakhstan, 2009-2018

Regions	Incidence							M/I	Mortality				
	per 100,000				р	Т_up/down_, %*	R^2^		per 100,000		р	Т %	R^2^
	Mean	m	95% CI						Mean	95% CI			
Pavlodar	51.5	1.7	48.2	54.9	0.745	+0.1	0.0019	0.33	16.9±0.9	15.2-18.6	0.010	−3.8	0.5614
Aktobe	38.2	1.2	35.8	40.5	0.035	+0.3	0.0077	0.33	12.8±0.7	11.3-14.2	0.072	−3.2	0.3271
Almaty city	61.9	2.9	56.2	67.6	0.207	+0.8	0.0277	0.31	19.2±1.0	17.3-21.1	0.006	−4.2	0.7005
East Kazakhstan	44.6	0.9	42.9	46.3	0.368	+1.2	0.2105	0.33	14.8±1.3	12.3-17.3	0.000	−7.5	0.7707
Astana city	61.2	2.3	56.7	65.8	0.624	+1.4	0.1334	0.31	19.3±1.1	17.1-21.4	0.219	−1.1	0.0387
Zhambyl	31.4	0.9	29.7	33.1	0.134	+1.4	0.4949	0.48	15.0±0.9	13.2-16.8	0.005	−2.6	0.1821
North Kazakhstan	47.5	1.1	45.3	49.7	0.048	+1.5	0.4097	0.26	12.4±0.9	10.6-14.1	0.002	−3.8	0.2854
Kyzylorda	30.4	0.8	28.8	32.0	0.579	+1.7	0.4137	0.39	11.7±0.9	10.1-13.4	0.262	−2.5	0.1182
Kostanay	43.1	2.0	39.2	47.1	0.053	+1.7	0.1449	0.29	12.7±0.8	11.2-14.2	0.027	−5.5	0.8547
Kazakhstan	42.6	1.0	40.7	44.5	0.000	+2.0	0.7277	0.32	13.9±0.6	12.7-15.0	0.000	−4.0	0.9218
South Kazakhstan	30.1	1.2	27.8	32.3	0.135	+2.7	0.4978	0.36	10.8±0.3	10.1-11.4	0.278	−0.8	0.0730
Akmola	40.8	1.7	37.4	44.2	0.069	+2.8	0.4149	0.36	14.6±0.8	13.0-16.3	0.034	−4.3	0.5693
West Kazakhstan	42.4	2.1	38.4	46.5	0.000	+3.2	0.4065	0.3	12.9±0.7	11.6-14.2	0.011	−4.0	0.6294
Karaganda	47.9	1.8	44.3	51.5	0.000	+3.2	0.6787	0.27	13.0±1.1	10.8-15.1	0.000	−7.4	0.7792
Almaty	33.7	2.2	29.3	38.0	0.001	+3.6	0.2827	0.32	10.8±0.5	9.8-11.8	0.008	−3.8	0.669
Atyrau	33.3	1.9	29.6	37.0	0.015	+4.0	0.4740	0.41	13.7±0.9	12.0-15.4	0.948	+1.7	0.0674
Mangistau	34.3	3.1	28.3	40.3	0.002	+6.1	0.4303	0.31	10.5±1.2	8.2-12.9	0.192	−5.4	0.2351

*The table is built taking into account the sorting from A to Z of the general growth.

## Discussion

Generally, during the study period, the number of patients with BC increased by 42.1% from 3,272 to 4,648 cases, which turned out to be higher than predicted according to the component analysis – 3,980. Herewith, the proportion of patients under 50 years of age was 27.4% and 72.6% in women over 50 years and older. A similar pattern was observed in the study of Kamińska and co-authors (Kamińska M et al., 2015), where BC was most common in women during menopause, 80% of cases of detection of the disease among women aged 50 years and older.

In Kazakhstan, there was a global trend in the incidence of BC. The results of the study showed that Kazakhstan (CR - 45.4 and ASR – 42.6 per 100,000 female population) refers to regions with an average incidence rate, countries such as Eritrea (42.1), Djibouti (42.4), the Republic of Moldova (42.6) and Qatar (42.7) (Ferlay et al., 2020). Whereas according to IARC data the incidence in Kazakhstan is indicated as 37.1

According to IARC (Ferlay et al., 2020), the highest standardized incidence rates per 100,000 female population were found in Luxembourg (99.8), the Netherlands (100.9), Belgium (113.2) and the Netherlands (182.9), while the lowest ones were found in Bhutan (5.0) and the Gambia (6.9). Early detection and different risk factors may explain the differences in BC variability worldwide.

The analysis of 95% CI of age-related indicators of incidence up to 60 years shows that they did not overlap, i.e. the difference is statistically significant (p<0.05) and the formation of indicators was influenced by various causal factors. After 75 years of age, 95% of the CI incidence rates of the studied ages did not overlap also. Age-related indicators of incidence show a unimodal increase, with a peak of incidence in 65-69 years – 167.2^0^/_0000_. The peak incidence of BC occurs at the end of postmenopausal age. This is most likely because the risk of BC diagnosis increases in women using hormone replacement therapy and rises with increasing duration of use. This effect diminishes after discontinuation of hormone replacement therapy and largely (if not completely) disappears after about 5 years, which should be considered with respect to the benefits and risks associated with this hormone treatment (Sood et al., 2014).

The rate of increase in the incidence of BC in the age group of 15-19 years, which is T_g_=+6.9, is very high and causes concern. Also, the high rates and high rate of growth in the incidence of women of fertile age (30-44 years T_g_=+2.4; R^2^=0.7138) indicates that in our republic, BC is increasingly covering young women. A more interesting aspect is that women with familial BC often develop this aggressive disease at an earlier age. This fact undoubtedly adds additional complexity and heterogeneity to the genetic landscape of BC in young women (Lianos et al., 2013; Odle, 2017; Zavala et al., 2019; Copur, 2019) and requires targeted study in Kazakhstan. A significant increase in BC cases in young women is very important, because the behavior of these tumors in most cases is more aggressive compared to older women. Available data indicate that BC in young women is a significant burden in developing countries compared to developed countries, and that a disproportionate number of young women die each year due to this type of cancer (Reyna and Lee, 2014; da Costa et al., 2017; Sancho-Garnier and Colonna, 2019; Feng et al., 2019; Azamjah et al., 2019).

In Kazakhstan, mammological screening is a mammography of both mammary glands in 2 projections, a “double reading” of mammograms by radiologists at the level of an oncological dispensary. A precondition for conducting a “double reading” of mammograms is the interpretation of mammograms by two radiologists independently of each other, in the future, the diagnosis of refinement – targeted mammography, breast ultrasound, biopsy. The screening interval is 1 every 2 years. The target group consists of women aged 40-70 years. In our study, we provided information on the target group, considering the age group of 40-69 years, since the accounting, registration and formation of reporting forms takes into account the age groups 0-4, 5-9, 10-14...65-69, 70-74 years, etc. (described in the materials and methods). So, what changes in BC incidence have occurred in the target group in Kazakhstan? The number of women aged 40-69 in 2009 was 29.9% of the total female population and in 2018 was 31.7%. The female population aged 40-69 increased by 20.0% during the study period. During the study period, 75.0% of the total number of new cases of BC were registered in the target group, and the incidence in this age group increased over time. Basically, these changes occurred because of changes that occurred in the age groups of 40-44 years (T_g_=+2.8%; R^2^=0.5179) and 65-69 years (T_g_=+6.38%; R^2^=0.5179), where the growth rate and the value of the approximation confidence were more pronounced. In the age groups of 50-54 years and 55-59 years, the trends in incidence were not pronounced, which should be paid attention to.

In an ideal scenario, screening can detect potentially fatal BC before it causes symptoms, i.e. in situ (before clinical symptoms occur). Detection of disease at an early stage would allow the best possible treatment and would mean fewer deaths from BC (Esserman et al., 2009; Bonsu and Ncama, 2019; Yuan et al., 2019; Cardoso et al., 2019). This has been the premise behind screening programs, but increasingly, researches show that the picture is much more complicated. BC takes many forms- some are slow-growing and harmless, and some are very aggressive and deadly, which grow and spread quickly. Because a screening mammogram is a snapshot in time, it is more likely to catch a slow-growing cancer than a fast-growing one (Welch and Black, 2010; Huang et al., 2017; Seely et al., 2018; Tagliafico et al., 2020; Tran et al., 2021). In other words, it leads to overdiagnosis because of its tendency to detect cancers that are unlikely to be malignant. It should be noted that in our studies we indicated that the incidence of BC in Kazakhstan increases in stage I-II, and decreases in stage III and IV (Tautayev et al., 2019; Igissinov et al., 2020), but no studies were conducted at the regional level.

The results of the component analysis indicate that in the republic, the incidence of BC in the entire female population is increasing mainly due to the influence of such a factor as the risk of acquiring illness (∑Δ_R_=+6.82^0^/_0000_), and the increase in patients due to this criterion was also significant in the republic (Δ_R_=+41.0%). Herewith, in the target group, the impact of the risk of acquiring illness on the incidence of BC was more pronounced (∑Δ_R_=+14.34^0^/_0000_), but the increase in the number of patients in this group (40-69 years) was more affected by the increase in the population (Δ_P_=+43.9%). Given that the “risk of acquiring illness” refers to the whole range of reasons that can lead to an increase, decrease or stabilization of incidence rates, we assume that screening has influenced the increase in the number of patients and morbidity in BC in the whole country. The formation and influence of the factors above were not the same in the regions. Thus, the analysis of incidence by region in the entire female population showed that the “risk of acquiring illness” component had no effect in the Pavlodar region (∑Δ_R_=−0.85^0^/_0000_), Astana city (∑Δ_R_=−3.59^0^/_0000_) and Almaty city (∑Δ_R_=−4.16^0^/_0000_). For the target group, this component had no impact in East Kazakhstan (∑Δ_R_=−3.08^0^/_0000_) and Pavlodar (∑Δ_R_=−4.96^0^/_0000_) regions, as well as in Almaty city (∑Δ_R_=−19.30^0^/_0000_) and Astana city (∑Δ_R_=−20.75^0^/_0000_). In addition, it was found that the growth of patients in these regions was not associated with the risk of acquiring illness: Astana city (Δ_R_=−18.2^0^/_0000_), East Kazakhstan region (Δ_R_=−19.7%), Pavlodar region (Δ_R_=−26.3%), Almaty (Δ_R_=−56.6%). Certain questions arise, why did these changes occur? Have the risk factors stopped influencing? Have preventive measures been improved? Are the changes related to accounting and registration? All this requires further in-depth and focused study. The obtained data indicate the presence of certain problems in the organization of anti-cancer measures, including screening.

Alongside this, there are regions where the increase in incidence was due to the greater influence of the “risk of acquiring illness” component. Thus, in the Mangystau region, the increase was 49.25^0^/_0000_ and it was due to the influence of the risk of acquiring illness ∑Δ_R_=+46.29^0^/_0000_, i.e. the contribution to the formation of the indicator was 94.0%. 

The same changes were in South Kazakhstan (∑Δ_R_=+13.83^0^/_0000_ – 74.3%), North Kazakhstan (∑Δ_R_=+26.02^0^/_0000_ – 78.2%), Atyrau (∑Δ_R_=+32.07^0^/_0000_– 78.9%), Almaty (∑Δ_R_=+15.85^0^/_0000_ – 79.9%), Aktobe (∑Δ_R_=+25.62^0^/_0000_ – 81.9%), Karaganda (∑Δ_R_=+52.08^0^/_0000_ – 86.6%), West Kazakhstan (∑Δ_R_=+53.42^0^/_0000_ – 87.2%) and Kyzylorda (∑Δ_R_=+13.24^0^/_0000_ – 87.6%) regions. The above has influenced the formation of the general trend in the republic as a whole and is assessed as a positive result of anti-cancer measures in breast cancer, including screening.

Unfortunately, it was not possible to compare our results with the literature data due to the lack of research in this direction.

An important criterion for evaluating anti-cancer measures is the analysis of mortality rates. Thus, standardized mortality rates decreased in almost all regions of Kazakhstan, except for the Atyrau region, where the trend rate tended to increase (T_g_=+1.7%), but the value of the approximation was not significant (R^2^=0.0674).

Thus, the conducted component analysis indicates that there are positive results of the influence of mammological screening in the country, while the influence of other exogenous and endogenous factors is not excluded. The obtained results are recommended for monitoring and evaluating anti-cancer measures in the republic.

## Author Contribution Statement

AT, ZT, AJ, GA – Collection and preparation of data, primary processing of the material and their verification.AT, ZB, NI – Statistical processing and analysis of the material, writing the text of the article (material and methods, results). AT, ZT, GI, AK – Writing the text of the article (introduction, discussion). NI, MM, BT – Concept, design and control of the research, approval of the final version of the article. All authors approved the final version of the manuscript.

## Conflict of interest

The authors declare that they have no competing interests; neither financial nor non-financial interests.
